# Fatigue, Polyuria, and Hidden Uveitis: A Case of Tubulointerstitial Nephritis and Uveitis Syndrome Diagnosed in Primary Care

**DOI:** 10.7759/cureus.89245

**Published:** 2025-08-02

**Authors:** Markus Kerner, Julia Todoroff, Christian von Schnakenburg, Kerstin Amann, Julian Müller-Kühnle

**Affiliations:** 1 General Practice, Dr. Kerner's General Medical Practice, Neuffen, DEU; 2 Pediatrics and Adolescent Medicine, Klinikum Esslingen, Esslingen, DEU; 3 Nephropathology, Friedrich-Alexander University Erlangen-Nürnberg, Erlangen, DEU; 4 General Internal Medicine and Nephrology, Robert Bosch Krankenhaus, Stuttgart, DEU

**Keywords:** adolescent renal disease, primary care diagnosis, tinu syndrome, tubulointerstitial nephritis, uveitis

## Abstract

Tubulointerstitial nephritis and uveitis (TINU) syndrome is a rare autoimmune condition primarily affecting adolescents. Diagnosis is frequently delayed due to the nonspecific and temporally dissociated presentation of renal and ocular symptoms. We report the case of a 15-year-old girl who presented to her general practitioner with fatigue, polyuria, and recent weight loss. Laboratory workup revealed acute kidney injury with elevated serum creatinine and a tubular pattern of proteinuria, prompting timely referral to a pediatric hospital. Renal biopsy confirmed acute, non-granulomatous tubulointerstitial nephritis. Despite lacking ocular complaints, ophthalmologic examination revealed bilateral anterior uveitis, confirming the diagnosis of TINU syndrome.

Notably, symptom onset followed the initiation of sertraline, suggesting a potential drug-induced trigger. The patient responded rapidly to high-dose oral corticosteroids, with full recovery of renal function and general well-being. Mild neuropsychiatric side effects occurred during treatment but resolved without intervention. Follow-up was coordinated through pediatric nephrology and ophthalmology.

This case highlights the role of primary care in the early recognition of rare systemic diseases. Tubular protein markers proved valuable in characterizing the renal injury pattern. In adolescents with unexplained renal dysfunction and constitutional symptoms, TINU should be part of the differential, even in the absence of visual complaints. Recent medication changes should raise suspicion for drug-induced immune-mediated nephritis.

## Introduction

Tubulointerstitial nephritis and uveitis (TINU) syndrome is a rare autoimmune disorder characterized by the concurrent involvement of the kidneys and eyes, most commonly presenting as bilateral, non-granulomatous anterior uveitis [1-3]. First described in 1975 [4], the condition predominantly affects adolescents and young adults, with a median age of onset around 14-15 years [5,6]. A distinct female predominance has been reported, with female-to-male ratios ranging from 3:1 to 5:1 [6,7].

Although only a few hundred cases have been published to date, the true prevalence of TINU is likely underestimated, particularly outside of ophthalmologic or nephrologic specialty settings [2,8]. It is estimated to account for approximately 2% of all uveitis cases [9], with most diagnoses occurring in pediatric populations, although adult-onset presentations have also been documented [10,11].

The exact pathogenesis remains incompletely understood. Current evidence suggests an autoimmune response directed against shared antigens in renal tubules and ocular structures [7], modulated by genetic predisposition, particularly specific human leukocyte antigen (HLA) haplotypes [12], and environmental triggers such as infections or medications [13-15].

Clinical presentation is heterogeneous and often nonspecific [2,16]. Renal manifestations may include fatigue, flank discomfort, polyuria, or signs of acute kidney injury [2,8], while ocular symptoms typically consist of bilateral anterior uveitis with pain, photophobia, or visual disturbances [2,16]. Importantly, some patients remain asymptomatic, and uveitis may be discovered incidentally on ophthalmologic examination [17].

Renal and ocular symptoms can occur simultaneously or sequentially, which may delay diagnosis [16,18]. Uveitis is frequently more persistent or prone to relapse than the renal component [2].

Diagnosis of TINU syndrome relies on clinical suspicion, laboratory findings, and, when feasible, histologic confirmation via renal biopsy. Laboratory abnormalities usually include elevated serum creatinine, mild proteinuria, and increased levels of tubular markers such as alpha-1-microglobulin (α1M) and urinary beta-2-microglobulin (β2M) [2,19]. Renal biopsy remains the diagnostic gold standard to confirm acute interstitial nephritis and to distinguish it from other causes of kidney injury, such as glomerulonephritis or antineutrophil cytoplasmic antibody (ANCA)-associated vasculitis [20]. Systemic conditions such as sarcoidosis, systemic lupus erythematosus (SLE), and other autoimmune disorders must be carefully excluded.

According to Mandeville et al., TINU syndrome can be classified as definite when both histologic or clinical evidence of acute interstitial nephritis and typical uveitis are present [6]. Cases are considered probable or possible if clinical features are incomplete or atypical.

A multidisciplinary diagnostic approach is essential, integrating clinical assessment [2], tubular protein markers [3,21], renal histopathology [2,21], and the exclusion of systemic disease [2,3,7].

Systemic corticosteroids are the mainstay of therapy [2,22,23], while immunosuppressants (e.g., methotrexate and azathioprine) or biologic agents (e.g., TNF-α inhibitors) may be required in refractory cases [2,24]. The overall prognosis is favorable, although relapses of uveitis are more frequent than renal relapses [23-25], and adults are at greater risk of progression to chronic kidney disease [2].

This case illustrates how clinical vigilance in primary care can facilitate the early recognition of TINU syndrome in an adolescent presenting with nonspecific systemic and renal symptoms.

## Case presentation

A 15-year-old girl (weight: 82 kg, height: 167 cm, body mass index (BMI): 29.4 kg/m²) presented to our primary care practice with complaints of persistent fatigue and increased urinary frequency over several weeks. She also reported an intentional weight loss of approximately 13 kg over recent months due to prolonged dietary restriction during remission of a previously diagnosed eating disorder. There was no history of fever, rash, joint pain, or gastrointestinal symptoms.

Her medical history included depression, for which she had started sertraline therapy four weeks prior to presentation. No other medications were being taken. On physical examination, the patient appeared mildly pale but was hemodynamically stable. There was no peripheral edema, rash, lymphadenopathy, or visible ocular involvement. Cardiopulmonary and abdominal examinations were unremarkable.

Initial laboratory testing revealed markedly impaired renal function with a serum creatinine level of 3.3 mg/dL and urea of 67 mg/dL. Mild anemia (hemoglobin: 11.8 g/dL) and elevated ferritin (282 µg/L) were also present. Inflammatory markers, including C-reactive protein (CRP) and white blood cell count, were within normal limits. Urinalysis showed proteinuria (100 mg/dL), glucosuria (300 mg/dL), microscopic hematuria (~50 erythrocytes/μL), and microalbuminuria (~50 mg/L). An overview of baseline laboratory parameters is provided in Table 1.

**Table 1:** Initial blood and urine parameters at first presentation Laboratory results indicated impaired renal function, mild anemia, elevated ferritin levels, and abnormal urinalysis with proteinuria, glucosuria, hematuria, and microalbuminuria. Reference ranges reflect standard pediatric laboratory values for adolescent females. CRP: C-reactive protein

Parameter	Result	Reference range
Serum creatinine	3.3 mg/dL	0.4-0.9 mg/dL
Urea	67 mg/dL	10-40 mg/dL
Hemoglobin	11.8 g/dL	12-16 g/dL
Ferritin	282 µg/L	13-150 µg/L
CRP	Normal	<5 mg/L (or laboratory-specific)
White blood cell count	Normal	4-10 × 10^9^/L
Urine-specific gravity	1.020	1.005-1.030
Proteinuria	100 mg/dL	Negative
Glucosuria	300 mg/dL	Negative
Microscopic hematuria	~50 erythrocytes/μL	<5 erythrocytes/μL
Microalbuminuria	~50 mg/L	<30 mg/L
Nitrites (urine)	Negative	Negative
Leukocytes (urine)	Negative	Negative

Given the unexpected renal impairment in an otherwise healthy adolescent, the patient was directly referred to a regional pediatric hospital for further evaluation. Renal ultrasonography revealed normal kidney size and echotexture without evidence of hydronephrosis, nephrocalcinosis, or structural abnormalities. An extended autoimmune panel, including antinuclear antibodies (ANA), ANCA, anti-glomerular basement membrane (anti-GBM) antibodies, and complement levels, was unremarkable.

Spot urine analysis demonstrated a markedly elevated protein-to-creatinine ratio (0.925 g/g; reference: <0.2 g/g), albumin (290 mg/g; reference: <20 mg/g), and α1-microglobulin (307 mg/g; reference: <14 mg/g). The disproportionate increase in α1-microglobulin relative to albumin strongly indicated a tubular pattern of proteinuria, supporting the diagnosis of isolated tubulointerstitial injury. This finding is consistent with published diagnostic schemes that distinguish glomerular from tubular protein loss [17].

On the third day after hospital admission, serum creatinine peaked at 3.59 mg/dL (317 µmol/L), corresponding to an estimated glomerular filtration rate (eGFR) of 17.5 mL/minute/1.73 m^2^, consistent with Kidney Disease: Improving Global Outcomes (KDIGO) stage 3 acute kidney injury. Despite the marked renal impairment, urine output remained clinically adequate. Arterial blood gas analysis at the time showed normal pH (7.39), potassium (4.4 mmol/L), and bicarbonate levels (24 mmol/L), without evidence of metabolic acidosis.

**Figure 1:**
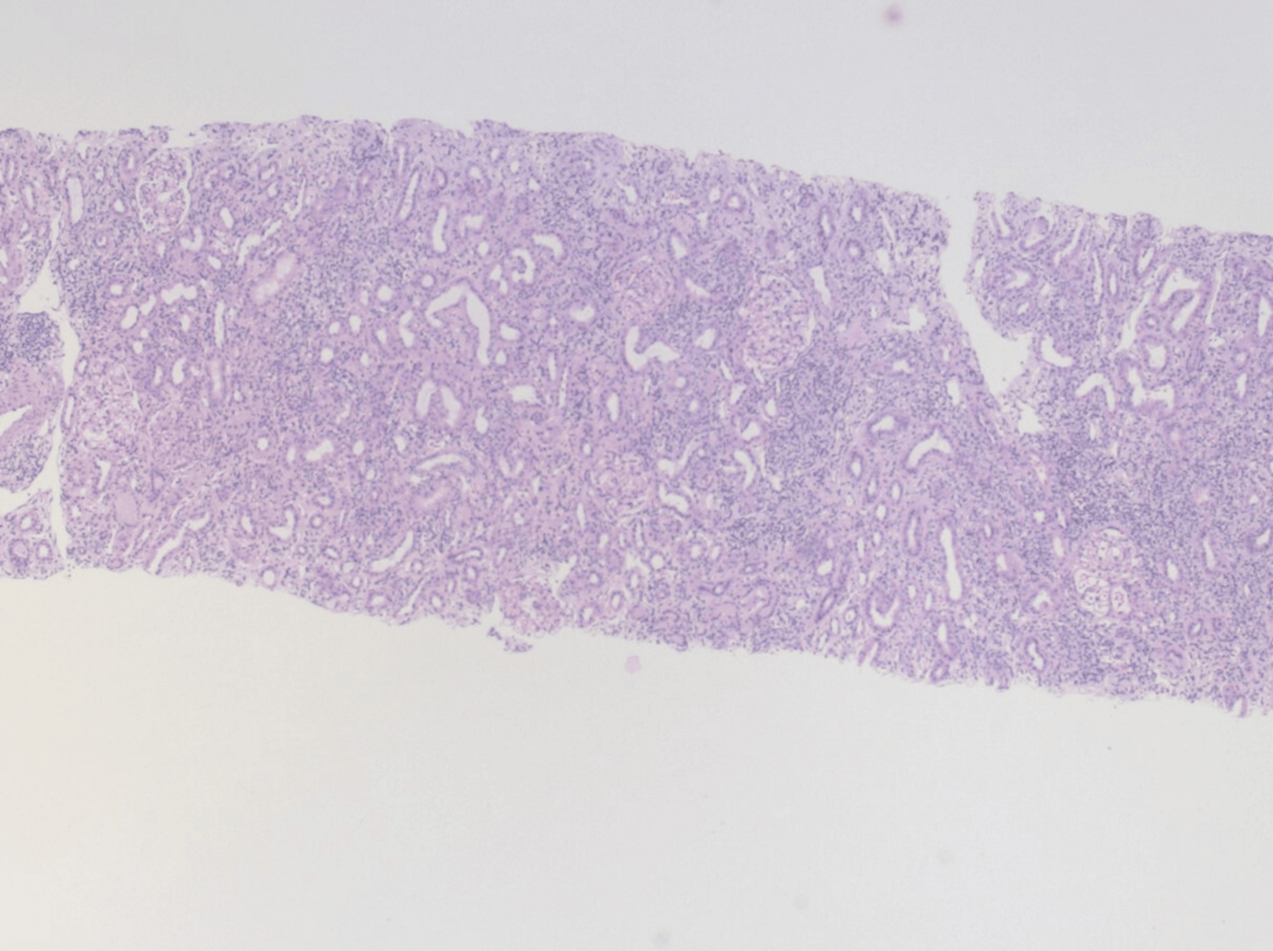
Low-power histologic image (5× magnification) of the renal cortex H&E stain showing diffuse interstitial inflammation with preserved glomeruli and no evidence of glomerular involvement. H&E: hematoxylin and eosin

**Figure 2:**
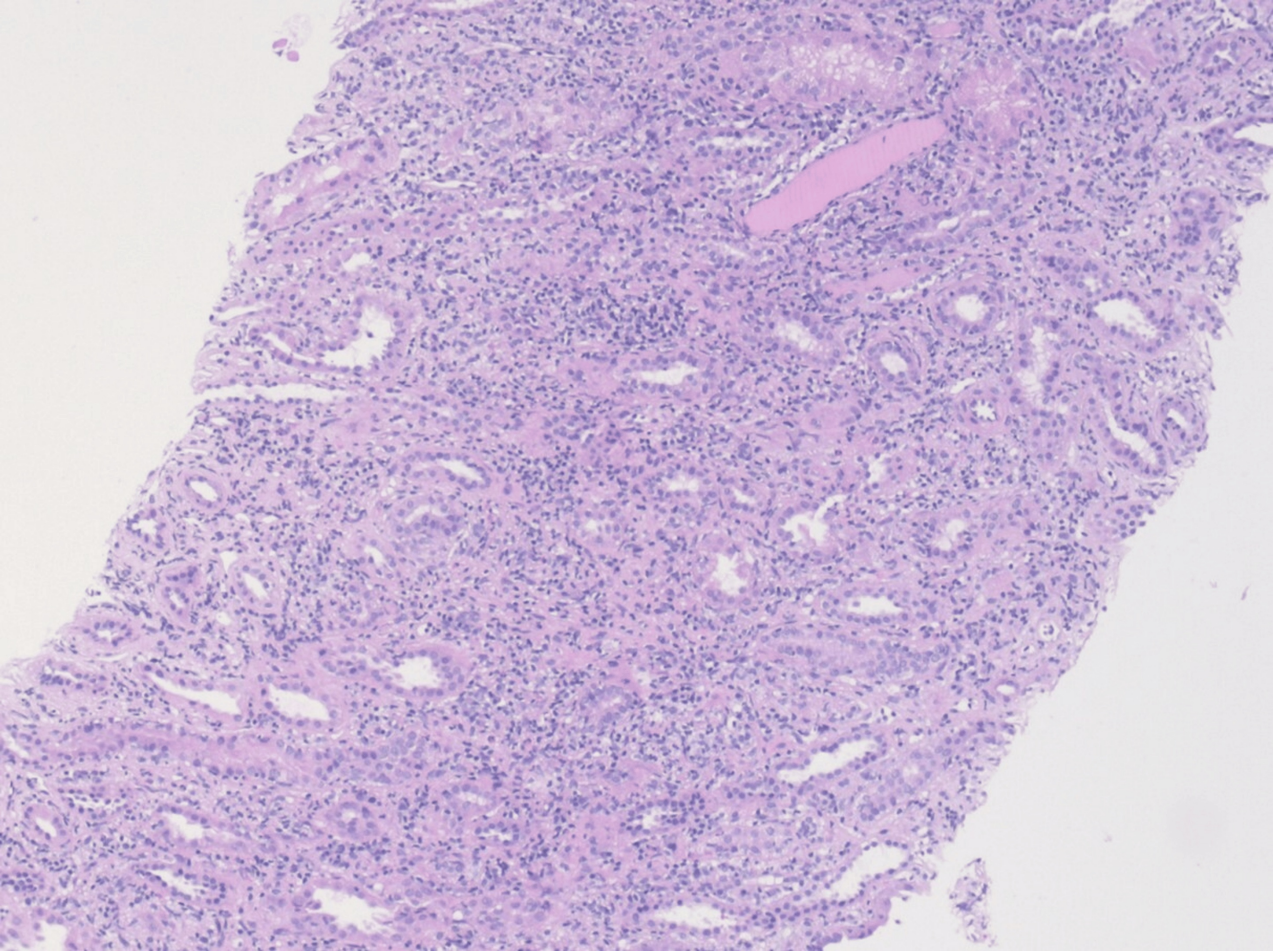
Medium-power histologic image (10× magnification) of the renal cortex H&E stain showing dense lymphoplasmacytic infiltration in the interstitium and evidence of tubular injury. No granulomas or vascular lesions are observed. H&E: hematoxylin and eosin

**Figure 3:**
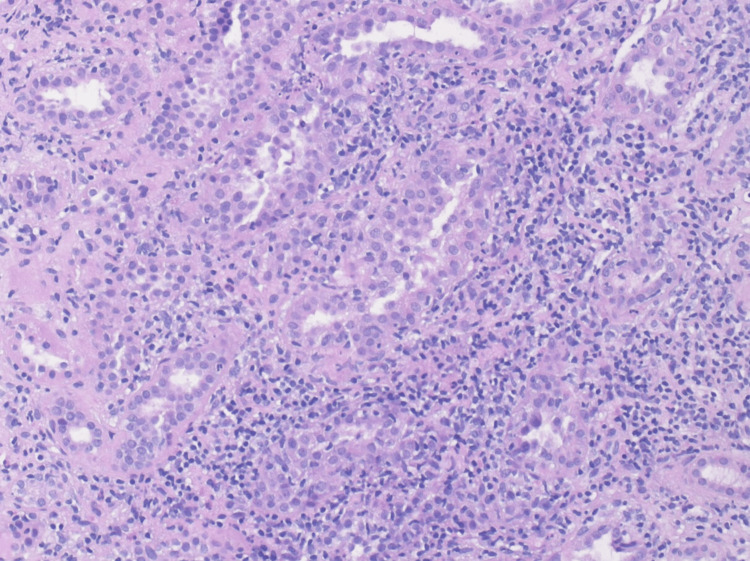
High-power histologic image (20× magnification) of the renal cortex H&E stain showing tubular epithelial damage with adjacent lymphocytic interstitial inflammation. No eosinophils or immune complex deposits are present. H&E: hematoxylin and eosin

Percutaneous renal biopsy revealed 29 glomeruli without evidence of glomerulosclerosis, immune complex deposition, or glomerulonephritis. The interstitium showed a diffuse, dense lymphoplasmacytic infiltrate without granuloma formation, accompanied by marked acute tubular injury. Tubular atrophy and interstitial fibrosis were each limited to less than 5% of the cortical area. No significant eosinophilia or vascular changes were noted. Immunofluorescence and electron microscopy were unremarkable, with no signs of complement- or immune complex-mediated glomerular disease. Overall, the findings were consistent with acute, non-granulomatous tubulointerstitial nephritis, highly compatible with TINU syndrome (Figures 1-3).

Following histologic confirmation, and in light of a possible drug-related etiology, sertraline was discontinued as a precautionary measure.

Ophthalmologic evaluation by an external specialist confirmed bilateral, non-granulomatous anterior uveitis on slit-lamp examination. Although photographic documentation was unavailable, the diagnosis was clearly recorded, and topical corticosteroids and mydriatics were initiated.

Initial ophthalmologic findings included active anterior chamber inflammation in the right eye, with visible Tyndall effect and anterior chamber cells, accompanied by mild conjunctival injection. A formal grading of anterior chamber cells (e.g., 1+ to 4+) was not provided in the external report. Corneal and retinal findings were unremarkable. The left eye showed no signs of inflammation. Best-corrected visual acuity was 1.0 in the right eye and 0.6 in the left; the cause of reduced visual acuity in the clinically unaffected left eye was not specified but may reflect pre-existing refractive error, amblyopia, or subclinical retinal changes.

On day 5 after hospital admission, histopathologic results from the renal biopsy became available, confirming acute tubulointerstitial nephritis. Based on these findings, systemic corticosteroid therapy with oral prednisolone was initiated at a weight-adjusted dose of 1 mg/kg/day, capped at a maximum of 80 mg/day in line with pediatric dosing recommendations, and administered under pediatric supervision. On the same day, a follow-up ophthalmologic examination revealed persistent anterior and vitreous inflammation in the right eye, with a reactive but narrowed pupil and suspected early posterior synechiae at the 5 o’clock position. The left eye now showed mild nasal blurring of the optic disc margin, raising suspicion for early papillitis or optic nerve edema. The full corticosteroid dose was maintained for two weeks before gradual tapering. During treatment, the patient experienced mild emotional lability and restlessness, which were managed conservatively without dose adjustment.

At two-week follow-up, serum creatinine had improved to 1.5 mg/dL. By week 4, creatinine had normalized to 1.18 mg/dL, and the patient reported a marked improvement in energy and well-being.

Urinary protein excretion also normalized: urine creatinine, 1.49 g/L; total urine protein, 144 mg/L; protein-to-creatinine ratio, 97 mg/g; and albumin-to-creatinine ratio, 8 mg/g. Follow-up results are summarized in Table 2.

**Table 2:** Follow-up urine parameters after four weeks of corticosteroid therapy All parameters had normalized, indicating resolution of tubular dysfunction and proteinuria.

Parameter	Result	Reference range
Urine creatinine	1.49 g/L	0.57-2.95 g/L
Total urine protein	144 mg/L	<150 mg/L
Protein-to-creatinine ratio	97 mg/g	<200 mg/g
Albumin-to-creatinine ratio	8 mg/g	<30 mg/g

Long-term multidisciplinary follow-up was initiated, including pediatric nephrology and ophthalmology. Given the patient’s nutritional history and recent weight loss, a full metabolic and nutritional assessment was performed and yielded no relevant abnormalities. Psychiatric status was considered stable under existing outpatient care, and no acute intervention was required during hospitalization. In view of the temporal association and the possibility of a drug-related cause, sertraline was not reintroduced during follow-up. Further psychiatric care, including medication management and psychosocial support, was continued in an outpatient setting.

## Discussion

TINU syndrome is a rare autoimmune condition that should be considered in adolescents presenting with nonspecific systemic symptoms and unexplained renal abnormalities [1,10]. While the majority of cases are diagnosed in nephrology or ophthalmology settings [1,3,6], this case underscores that timely recognition in primary care is possible, provided that general practitioners maintain a broad differential and act on clinical cues suggestive of systemic disease.

Our patient presented with fatigue, polyuria, and additional weight loss that was no longer intentionally induced, symptoms that, in the context of a history of depression and prior disordered eating, might easily have been attributed to psychosomatic causes. However, initial laboratory testing revealed significant renal impairment, prompting appropriate referral and further nephrologic and ophthalmologic evaluation. The final diagnosis was established through a combination of acute interstitial nephritis on biopsy and bilateral anterior uveitis on slit-lamp examination, as outlined in the diagnostic framework for TINU syndrome.

Notably, ocular involvement was not clinically apparent at first presentation. This is consistent with previous reports suggesting that uveitis may be mild, bilateral, and asymptomatic, underscoring the importance of proactive ophthalmologic assessment in patients with unexplained interstitial nephritis [1,25]. The temporally dissociated onset of renal and ocular findings remains one of the principal challenges in TINU diagnosis [14,16].

Another diagnostically relevant feature in this case was the use of tubular protein markers to distinguish between glomerular and tubular injury. The marked elevation of α1-microglobulin relative to albumin in spot urine analysis strongly supported a tubular pattern, aligning with diagnostic frameworks such as the Urine Protein Expert System (UPES) proposed by Lun et al. [24]. Such markers may provide both diagnostic and prognostic value in pediatric patients with acute kidney injury.

The temporal association between the initiation of sertraline therapy and the onset of interstitial nephritis in our patient raises the possibility of a drug-induced immune response. While selective serotonin reuptake inhibitors (SSRIs) are not among the most common causes of drug-induced AIN, case reports and pharmacovigilance data have occasionally implicated agents such as fluoxetine and sertraline [26,27]. Although causality in this case remains unproven, clinicians should remain aware of this potential association, especially in adolescents starting new psychotropic medications.

The patient responded rapidly to systemic corticosteroids, with marked improvement in renal function and overall well-being. Mild neuropsychiatric side effects, including emotional lability and restlessness, were noted during treatment, which is consistent with prior literature on corticosteroid-associated mood alterations in adolescents with psychiatric comorbidities [28,29]. These observations highlight the need for psychological monitoring and a multidisciplinary approach to care.

Long-term outcomes in TINU syndrome are generally favorable when the condition is identified early and managed appropriately. Nevertheless, uveitis tends to relapse more frequently than nephritis and may persist or reappear months to years after the initial episode [21-23]. Regular follow-up with both pediatric nephrology and ophthalmology remains essential.

Emerging evidence suggests that the degree of low-molecular-weight proteinuria may correlate with the time to renal recovery in pediatric TINU. In a recent cohort study, children with more pronounced tubular protein loss had a longer duration to full renal remission [30]. These findings emphasize the clinical value of integrating quantitative tubular markers into the diagnostic and monitoring process.

In summary, this case highlights three key clinical insights. TINU syndrome should be included in the differential diagnosis of adolescents presenting with unexplained renal dysfunction and constitutional symptoms, even in the absence of ocular complaints. Primary care physicians play a pivotal role in initiating early workup and specialist referral in systemic inflammatory conditions. Tubular protein markers such as α1-microglobulin are valuable tools for diagnosing and monitoring acute interstitial nephritis and should be part of the standard urine workup in pediatric patients.

## Conclusions

This case highlights the critical role of clinical vigilance and integrative diagnostic reasoning in primary care. Early recognition of a rare systemic condition such as TINU syndrome by a general practitioner enabled prompt referral, timely diagnosis, and effective treatment. In adolescents presenting with fatigue, polyuria, and unexplained renal dysfunction, TINU should be considered in the differential diagnosis, even in the absence of overt ocular symptoms. Recent initiation of potentially nephrotoxic medications should further raise clinical suspicion for drug-induced interstitial nephritis in this context.
